# Regularization and grouping *-omics* data by GCA method: A transcriptomic case

**DOI:** 10.1371/journal.pone.0206608

**Published:** 2018-11-01

**Authors:** Monika Piwowar, Kinga A. Kocemba-Pilarczyk, Piotr Piwowar

**Affiliations:** 1 Department of Bioinformatics and Telemedicine, Jagiellonian University–Medical College, Krakow, Poland; 2 Chair of Medical Biochemistry, Jagiellonian University Medical College, Krakow, Poland; 3 AGH University of Science and Technology, Faculty of Electrical Engineering, Automatics, Computer Science and Biomedical Engineering, Department of Measurements and Electronic, Krakow, Poland; Chuo University, JAPAN

## Abstract

The paper presents the application of Grade Correspondence Analysis (GCA) and Grade Correspondence Cluster Analysis (GCCA) for ordering and grouping *-omics* datasets, using transcriptomic data as an example. Based on gene expression data describing 256 patients with Multiple Myeloma it was shown that the GCA method could be used to find regularities in the analyzed collections and to create characteristic gene expression profiles for individual groups of patients. GCA iteratively permutes rows and columns to maximize the tau-Kendall or rho-Spearman coefficients, which makes it possible to arrange rows and columns in such a way that the most similar ones remain in each other’s neighbourhood. In this way, the GCA algorithm highlights regularities in the data matrix. The ranked data can then be grouped using the GCCA method, and after that aggregated in clusters, providing a representation that is easier to analyze–especially in the case of large sets of gene expression profiles. Regularization of transcriptomic data, which is presented in this manuscript, has enabled division of the data set into column clusters (representing genes) and row clusters (representing patients). Subsequently, rows were aggregated (based on medians) to visualise the gene expression profiles for patients with Multiple Myeloma in each collection. The presented analysis became the starting point for characterisation of differentiated genes and biochemical processes in which they are involved. GCA analysis may provide an alternative analytical method to support differentiation and analysis of gene expression profiles characterising individual groups of patients.

## Introduction

Modern high-throughput methods produce large volumes of -*omics* data. Efficient processing of such data, in conjunction with other available biomedical datasets, is one of the main challenges facing modern biostatisticians and bioinformatics experts [1]. To extract information from such datasets, multidimensional analysis is commonly applied. Classical statistical methods are adapted to process large volumes of data, or data may be preprocessed–with the use of biological knowledge–as a preliminary step in statistical processing pipelines [2] [3] [4]. It is also becoming more and more common to adopt an integrative approach based on specialied databases, with various configurations of analytical methods facilitating simple and efficient extraction of relevant information using custom analysis platforms [5] [6]. Nevertheless, in spite of the dynamic evolution of data analytics, the capabilities of existing IT frameworks lag behind the sheer volume of data sets produced by modern research tools.

This manuscript presents the Grade Correspondence Analysis (GCA) application, which is custom-tailored for transcriptomics data and addresses the aforementioned challenges. GCA exemplifies the rapidly expanding field referred to as *data mining* and constitutes an essential step towards the integration of statistics, data exploration, taxonomy and measurement theory, with continuous and discrete data treated in a similar manner [7]. The GCA process involves identifying regularities and dependencies between variables and observations and helps define data clusters in predictive analysis problems. Regularity metrics are used to subdivide each dataset into clusters, based on different monotonic models than in the source matrix. Grade analysis methods can be applied to search for trends (hidden structure), groups and outliers. GCA analysis has so far been successfully used in (a) identifying concentrations of vital elements (calcium, magnesium, zinc, iron and copper) and two toxic elements (lead and cadmium) in hair tissue (over 20 thousand subjects); (b) analysis of parliamentary election results in selected electoral circuits [8], and (c) processing of images where pixels are described by selected variables [9].

GCA may also constitute a valuable exploratory and analytical tool for -*omics* data, facilitating proper data classification and therefore increasing the accuracy of decisions related to, e.g. custom therapies for patients with specific transcriptomic profiles.

This paper discusses the results of GCA and Grade Correspondence-Cluster Analysis carried out on gene expression datasets obtained from Multiple Myeloma patients. The analysis resulted in a reduction in the dimensionality of input data while enabling patient records to be assigned to regular layers and uniform, well-ordered clusters. The outcome was a set of distinct patient groups and gene clusters with characteristic levels of expression, providing input for further analysis of biochemical pathways. The Multiple Myeloma example, presented here as a case study, shows how to isolate groups of patients (rows) and sets of genes (columns) from a large transcriptomics dataset to characterise patients with specific genetic profiles.

## Materials and methods

### Data analysis scheme

**Fig 1:**
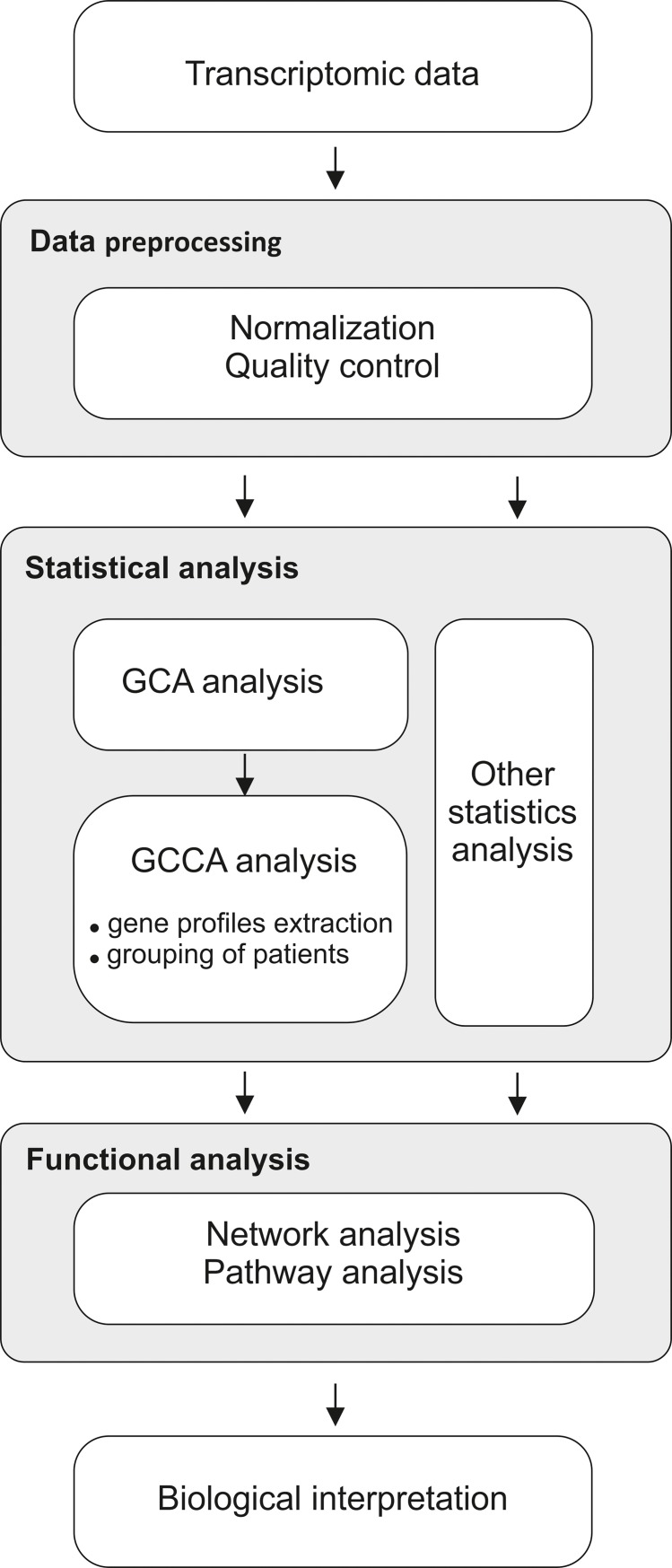
Data analysis workflow.

Data analysis workflow consists of the following stages (Fig 1):

- Data preprocessing. The data used for analysis must be subjected to quality analysis and normalisation to remove outliers and to allow comparison of samples.
- Statistical analysis (GCA and GCCA). The normalised data is sorted and grouped according to GCA and GCCA methodology, respectively.
- Functional analysis. The grouped data allows the analysis of a narrower number of data (in groups) regarding their e.g. function in biological processes.
- Biological Interpretation.
- The most important stage of the analysis is the interpretation of the results, supported by the results obtained from the app assessing the function that the products of the analysed genes meet.

The individual steps of the workflow are described below.

#### Microarray data

Gene expression data used in the study was publicly available and deposited at the NIH Gene Expression Omnibus (GEO) National Center for Biotechnology Information. The accession number of the source of data: GSE2658. The data concerned the U133 Plus 2.0 Affymetrix oligonucleotide microarray data from 256 newly diagnosed MM patients undergoing total therapy 2 (TT2), provided by the Donna D. And Donald M. Lambert Laboratory of Myeloma Genetics, the University of Arkansas for Medical Sciences, Little Rock, AR, USA [10]. The data set of 559 myeloma patients (GSE2658) is composed of a patient enrolled in two different therapies, total therapy 2 (TT2) and total therapy 3 (TT3). The microarray analysis was performed on malignant plasma cells at diagnosis of the disease and in consequence the gene expression profile analysed in the manuscript is not influenced by any kind of therapy. Thus, there is no difference in the expression profile between TT2 and TT3 group at starting point but the differences are expected once the treatment has been completed. Taking into consideration that the relation between particular expression profile and the clinical parameters as overall survival and progression free survival needs to be analysed separately for TT2 and TT3 group, the one of two groups, exactly the TT2, has been selected for analysis.

#### Pre-processing data

For background correction and normalisation of gene expression data the limma library [11] for the R, environment was used [12] [13]. To reduce the variability of log-ratios for low-intensity spots the 'normexp' background correction method was used, while to preserve comparability of distributions across samples the ‘quantile’ normalisation method was applied.

#### Statistical analysis: Grade Correspondence Analysis (GCA) and Grade Correspondence-Cluster Analysis (GCCA)

Data analysis proceeded with the use of the Grade Correspondence Analysis algorithm (GCA), which seeks regularities in data matrices and identifies correspondences between their rows and/or columns [14].

The GCA algorithm accepts a data matrix consisting of *n* rows (patients), each of which comprises k normalized nonnegative values (columns) which correspond to individual genes. This input is transformed into a matrix with dimensions n*k which can be formally treated as a probability matrix P_n*k_ = [p_ij_] for a two-dimensional distribution. Quantification of measure (3) yields, for each unit square, a nonnegative function called grade density. Subsequently, GCA iteratively permutates rows and columns in order to maximize either Kendall’s tau or Spearman’s rho, arranging them in such a way as to ensure that similar rows and/or columns are proximate to each other. For the bigger n*k tables permutations randomly rows and columns and reorders them to achieve a local maximum of the tau-Kendall (tau) or rho-Spearman (rho) coefficients is time-consuming and computationally demanding. In that cases, to reach a global maximum of tau or rho within a reasonable time, simulations are used, e.g. Monte Carlo. In effect, GCA uncovers regularities and monotonicity present in the input matrix, revealing hidden trends.

When the data structure is irregular and contains no strong monotonic dependencies, outlier detection may be applied to single out disruptive elements and subsequently identify regular subsets referred to as layers.

The subsequent phase, called Grade Correspondence-Cluster Analysis (GCCA), involves decomposition of each regular data layer produced by GCA into more uniform subsets. At this point, segmentation becomes bidirectional and yields segments consisting of successive, adjacent cases (rows or records) as well as variables (columns). The number of clusters is arbitrarily defined. Clusters are formed by variance coefficients, maximising the variance between each pair of groups, i.e. between objects formed by aggregating all elements which comprise each cluster. The target number of clusters is determined by commonly used scree plots.

The data can be graphically represented as a heatmap.

Grade Correspondence-Cluster Analysis also enables a reduction in data volume by aggregating adjacent rows and columns within each cluster, or by singling out representative cases for each cluster.

Grade analysis is based on the so-called *grade transformation* [14] defined for the cumulative distribution function *F* of a random variable *X* as follows (1):

$$F^{*}(u,x)=\{\begin{array}{c} 1\\ \frac{u-F(x-)}{F(x+)-F(x-)}\\ 0 \end{array},\;\begin{array}{c} if\;F(x+)\leq u,\\ if\;F(x-)\leq u<F(x+)\\ if\;F(x-)>u \end{array}$$ (1)

u∈[0,1],x∈R

Where F(x+) is the right-handed limit while G(x-) is the left-handed limit of F at point x.

The grade transformation is, therefore, the only transformation of a random function through F which is independent of distribution type. It results in a uniform distribution over the unit range, i.e. F*(u) = u; u belongs to [0,1].

Assuming that the cumulative distribution function H(x,y) describes the joint distribution of the random vector H(X,Y); F(x) and G(y) are distribution functions which correspond to edge distributions X and Y; while F*(u,x) and G(v,y) are their corresponding grade transformations; the grade transformation of the cumulative distribution function H(2), given as

$$H^{*}(u,v)=\iint_{R^{2}}F^{*}(u,v)G^{*}(v,y)dH(x,y),$$ (2)

u,v∈[0,1],x,y∈R

Maps H onto an unambiguous two-dimensional copula H* referred to as the grade distribution (X,Y). Wherever the edge distribution functions F and G remain continuous this copula coincides with Sklar’s copula. [15].

The edge distributions for copulas are monotonous over the [0,1] range. The grade distribution is continuous; the grade density of vector (X,Y) is defined as the distribution density of its corresponding column. Coefficients which result from applying the aforementioned transformation to vector parameters are also referred to as grade coefficients.

When the distribution of (X,Y) is discrete, with a probability matrix P_n*k_

$$P_{n*k}=\left(P_{i,j}\right),\;i=1,2,\ldots \;n,j=1,2,\ldots \;k$$

grade density can be defined as follows (3):

$$h^{*}(u,v)=\frac{p_{ij}}{p_{i}\cdot p_{j}},\;(u,v)\in R_{\mathrm{ij}},\;R_{\mathrm{ij}}=[S_{i-1},S_{i})*[T_{j-1},T_{j})$$ (3)

Where S_i_ = p_1._+…..+p_i._, T_j_ = p_.1_+…..+p_.j_, S_0_ = T_0_ = 0, i = 1, … n, j = 1, … k

$$p_{i.}=\Sigma_{1}{}^{k}p_{i,j}\;p_{.j}=\Sigma_{1}{}^{n}p_{i,j}$$

is the density of the two-dimensional distribution with monotonous edges, uniform over each rectangular region R_ij_ which belongs to the unit square.

When X and Yare independent, p_ij_ = p_i._p_.j_, grade density becomes equal to 1. Accordingly, grade density at point (u,v) may be interpreted as the measure of local overrepresentation of the distribution corresponding to pair (X,Y) with respect to the independent distribution (sharing the same edge values). Charting the grade density for the unit square, using color-coded values, results in the so-called overrepresentation map. Overrepresentation may adopt any nonnegative value; however, values from the [0,1) range are sometimes referred to as underrepresentation.

An important example of a parameter which is invariant to the (X,Y) grade transformation is the grade correlation coefficient, also known as Spearman’s rho: it directly maps to the grade distribution correlation coefficient (Spearman’s correlation coefficient for the copula) while retaining its value. Spearman’s rho is one of the nonparametric measures of a monotonic relationship between random variables.

For probability matrix P_n*k_ this coefficient is given by (4):

$$\rho^{*}(P_{n*k})=3\sum_{j=1}^{k}\sum_{i=1}^{n}(S_{i-1}+S_{i}-1)(T_{j-1}+T_{j}-1)p_{i,j}$$ (4)

Where p_i._, p_,j_, S_i_ and T_j_ are defined as above.

Another measure of the monotonic relationship between random variables which is invariant to the grade transformation is Kendall’s tau coefficient, which, for an arbitrary probability matrix P_n*k_, can be expressed as (5):

$$\tau (P_{n*k})=2\sum_{r=2}^{n}\sum_{i=1}^{r-1}\sum_{s=2}^{k}\sum_{j=1}^{s-1}\left(p_{ij}p_{rs}-p_{rj}p_{is}\right)$$ (5)

Both rho (6) and tau (7) may also be expressed as measures of variance of rows (or columns) in probability matrix P_n*k_ in the following manner [7]:

$$\rho^{*}(P_{n*k})=3\sum_{r=2}^{n}\sum_{i=1}^{r-1}\left(S_{r}+S_{r-1}-S_{i}+S_{i-1})p_{i*}p_{r*}ar(r:i\right)$$ (6)

$$\tau (P_{n*k})=2\sum_{r=2}^{n}\sum_{i=1}^{r-1}p_{i*}p_{r*}ar(r:i)$$ (7)

Where ar(r:i) is the value of the vector variance coefficient:

$$\left(\frac{p_{r1}}{p_{r*}},\frac{p_{r2}}{p_{r*}},\ldots ,\frac{p_{rn}}{p_{r*}}\right)\;\mathrm{and}\;\left(\frac{p_{i1}}{p_{i*}},\frac{p_{i2}}{p_{i*}},\ldots ,\frac{p_{in}}{p_{i*}}\right)$$

and is defined as (8):

$$ar(r:i)=\sum_{s=2}^{k}\sum_{j=1}^{s-1}\frac{\left(p_{ij}p_{rs}-p_{rj}p_{is}\right)}{p_{r*}p_{i*}}$$ (8)

Monotonic regularity is expressed by the so-called regularity index [16] (9):

$$reg=\frac{\tau_{\max(p)}}{\tau_{abs(P)}}$$ (9)

Where tau max is the peak value of τ over all possible permutations of rows and columns from P_n*k_, while tau abs (10) is defined as:

$$\tau_{abs}(P_{n*k})=2\sum_{r=2}^{n}\sum_{i=1}^{r-1}\sum_{s=2}^{k}\sum_{j=1}^{s-1}\left|p_{ij}p_{rs}-p_{rj}p_{is}\right|$$ (10)

and describes the total variance for all columns and rows in P_n*k_

This measure is also invariant to cupola transformation.

The basic tool of grade analysis is the GCA (Grade Correspondence Analysis) algorithm, which attempts to maximise regularity within a data matrix and also identify the strongest correlations between its rows and columns.

#### Functional analysis

To analyse and visualise the gene terms for large clusters of genes in a functionally grouped network the ClueGo (Cytoscape plug-in) was used [17]. The ClueGo improve biological interpretation of large lists of UP and DOWN regulated genes. It has implemented enrichment tests based on the hypergeometric distribution with Benjamini-Hochberg correction for multiple testing.

#### Comparision of GCA versus CUR and random results

The GCA results were compared with an additional way of analysis of gene expression and discriminant gene selection which was the CUR-based matrix decompositions method implemented in the rCUR package [18]. Based on the CUR methodology [19], 10% the probes being the of the whole dataset were identified (k = 4) (rCUR results). Then this subset was sorted by the GCA method and clustered for 6 clusters in a similar way to the whole original dataset. The rCUR results were compared to the original data. The GCA results were also compared with the randomly selected probes divided into six clusters.

## Results and discussion

### Grade Correspondence Analysis (GCA) results

**Fig 2:**
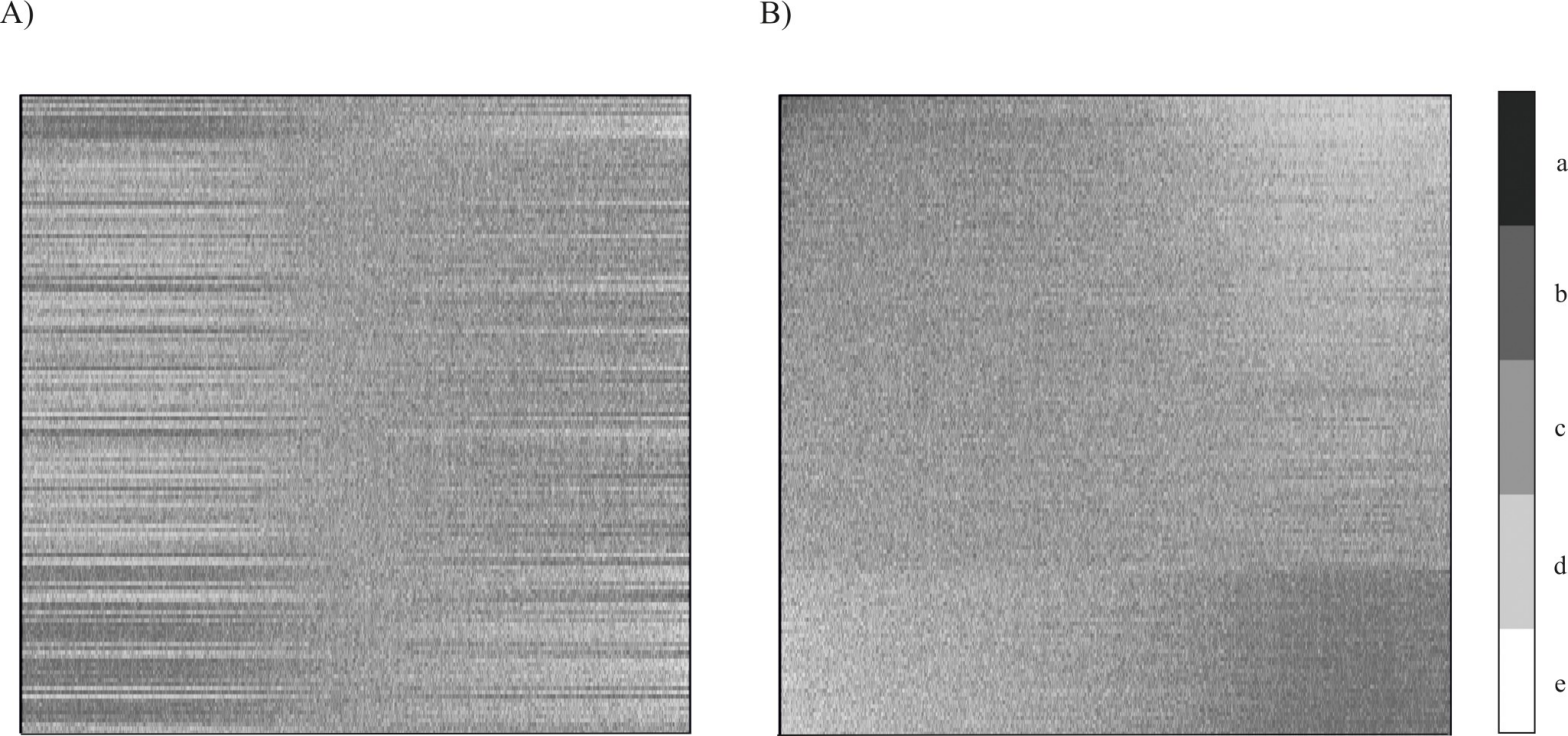
Gene expression maps for 54 677 probes in columns (genes) and 256 patients (rows). A) raw data distribution, before applying GCA; B) overrepresentation map revealing the dominant trend after GCA has been applied. Specific values are represented by shades of grey (with darker shades corresponding to greater values). The lighter the shading (d,e), the closer the value is to 0; the darker the shading (a,b), the greater the ratio; c–ideal representation.

By maximising the Rho gradation correlation, the GCA algorithms “sorts” the rows and columns of the input matrix. As a result, regular distribution of gene expression profiles (columns representing probes) for individual patients (rows) is obtained (Fig 2). This ordering is such that the “trend structure” shows up as darker shading running from the top left to the bottom right corner, concentrating at these opposite corners. It reveals dependencies whose strength is measured by global differentiation (peak value of the “rho” coefficient for the input dataset). The overrepresentation map is composed of 256 horizontal rows where each row represents a patient. 54 677 columns correspond to 54 677 variables (genes).

The value range is represented by shading. Darker areas mean “higher than expected” values in the expression matrix. In the presented case, the overrepresentation map obtained after using the GCA algorithm indicates a higher number of transcripts genes (columns) in areas marked by intense shading, and a lower number of transcripts in relation to the expected value in lightly shaded areas (Fig 2B). It can be seen that in some patients the expression of certain genes is stronger than in other patients.

### Grade Correspondence-Cluster analysis (GCCA)

**Fig 3:**
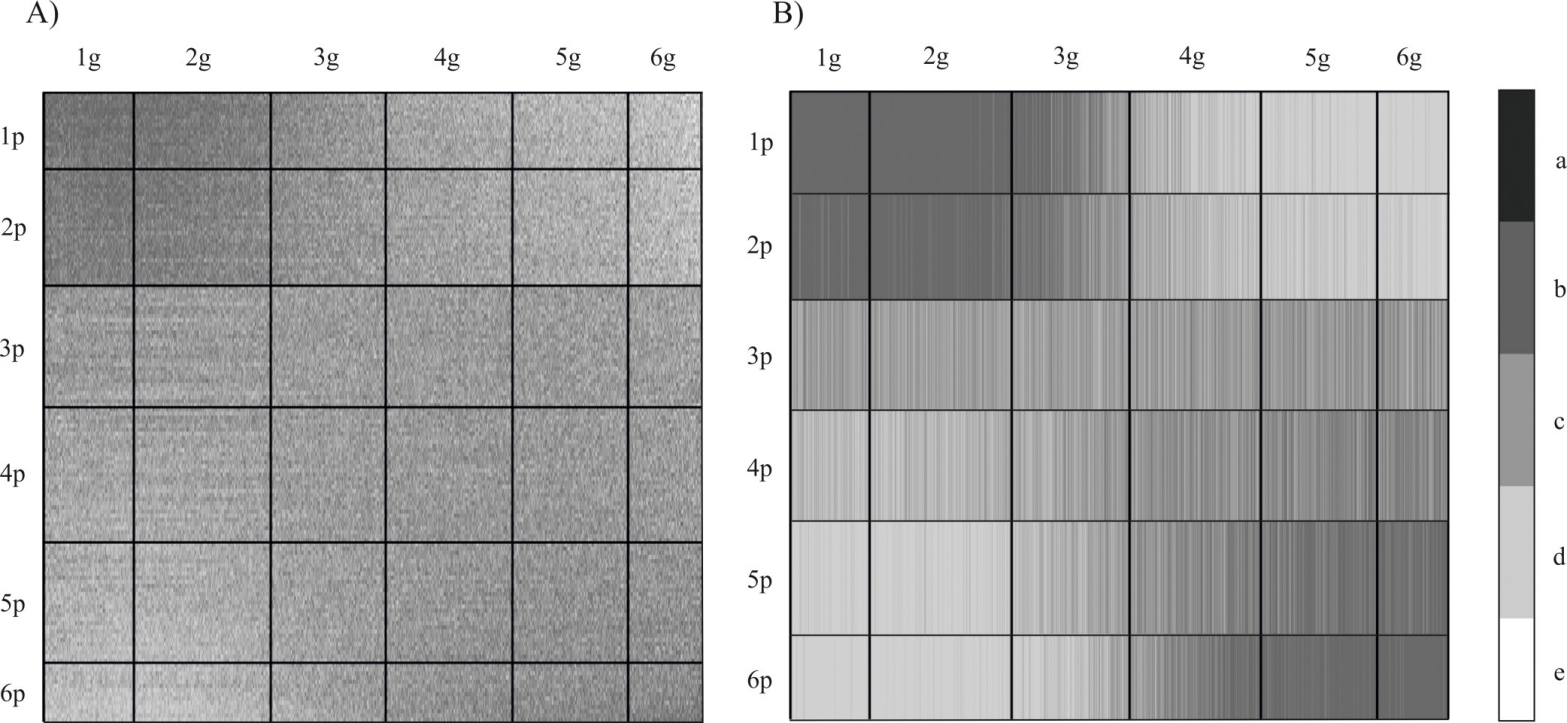
Overrepresentation maps of gene expression after GCCA A) clustering previously ordered data in rows and columns on six groups B) aggregation data in row clusters by median; bold vertical bars separate gene clusters (1p-6p); bold horizontal lines separate patient clusters (1g-6g). The lighter the shading (d,e), the closer the value is to 0; the darker the shading (a,b), the greater the ratio c–ideal representation.

To distinguish groups of patients, with the most extreme differences in gene expression profiles, cluster analysis was performed using the GCCA method. The group of patients was divided into six clusters. Similarly, in the case of genes, to differentiate groups of genes with a similar level of expression, they were also divided into six clusters (Fig 3A).

The data presented in Fig 3A is divided into six cluster groups, both horizontally (rows) and vertically (columns). These variable clusters are a permutation of the 54 677 columns representing genes (probes) and 256 rows previously ordered by GCA. As clusters are formed by computing variance coefficients and maximizing the variance between each pair of clusters, the resulting width of each cluster may vary.

**Fig 4:**
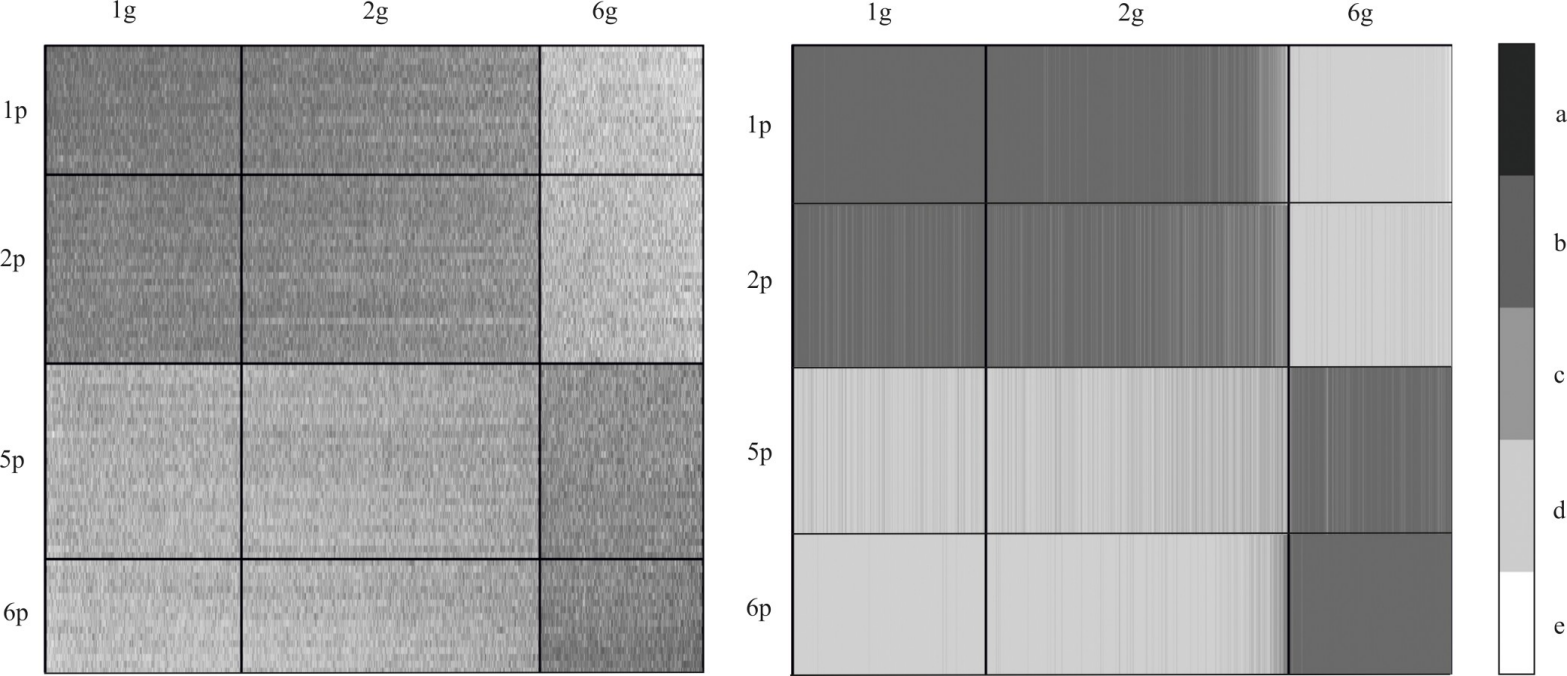
Overrepresentation map showing the most diverse clusters of genes (columns– 1g, 2g and 6g) and patients (rows aggregated by median– 1p, 2p, 5p and 6p). The lighter the shading (d,e), the closer the value is to 0; the darker the shading (a,b), the greater the ratio c–ideal representation.

In the next step, a median value was calculated for each cluster (Fig 3B). This approach increases the readability of the obtained result. Our focus then shifted to those representations for which the observed differences were the largest, i.e. they contained the level of the most differentiating genes expression. Visualization of data after GCCA revealed groups of patients (rows) whose expression profiles significantly differed from the expected values, i.e. they were significantly higher or lower (clusters 1/2 and 5/6 respectively). The clusters of genes (in columns) reveal genes whose expression was either higher or lower (clusters 1/2 and 6 respectively) depending on the group of patients (Fig 3B). Hence, the further analysis focused on patient clusters 1, 2 and 5, 6, while the central rows 3 and 4 were omitted (Fig 4). Gene clusters 3, 4 and 5 (columns) were similarly omitted, leaving only the strongly differentiated clusters 1, 2 and 6 (Fig 4).

#### Properties of over- or underexpressed genes

Gene expression among patients with Multiple Myeloma is not very diverse (expression profiles are similar). However, the results of GCA sorting allowed us to distinguish two groups of patients with slightly different profiles: the group represented by clusters 1 and 2, and another group consisting of clusters 5 and 6 (Fig 4).

**Fig 5:**
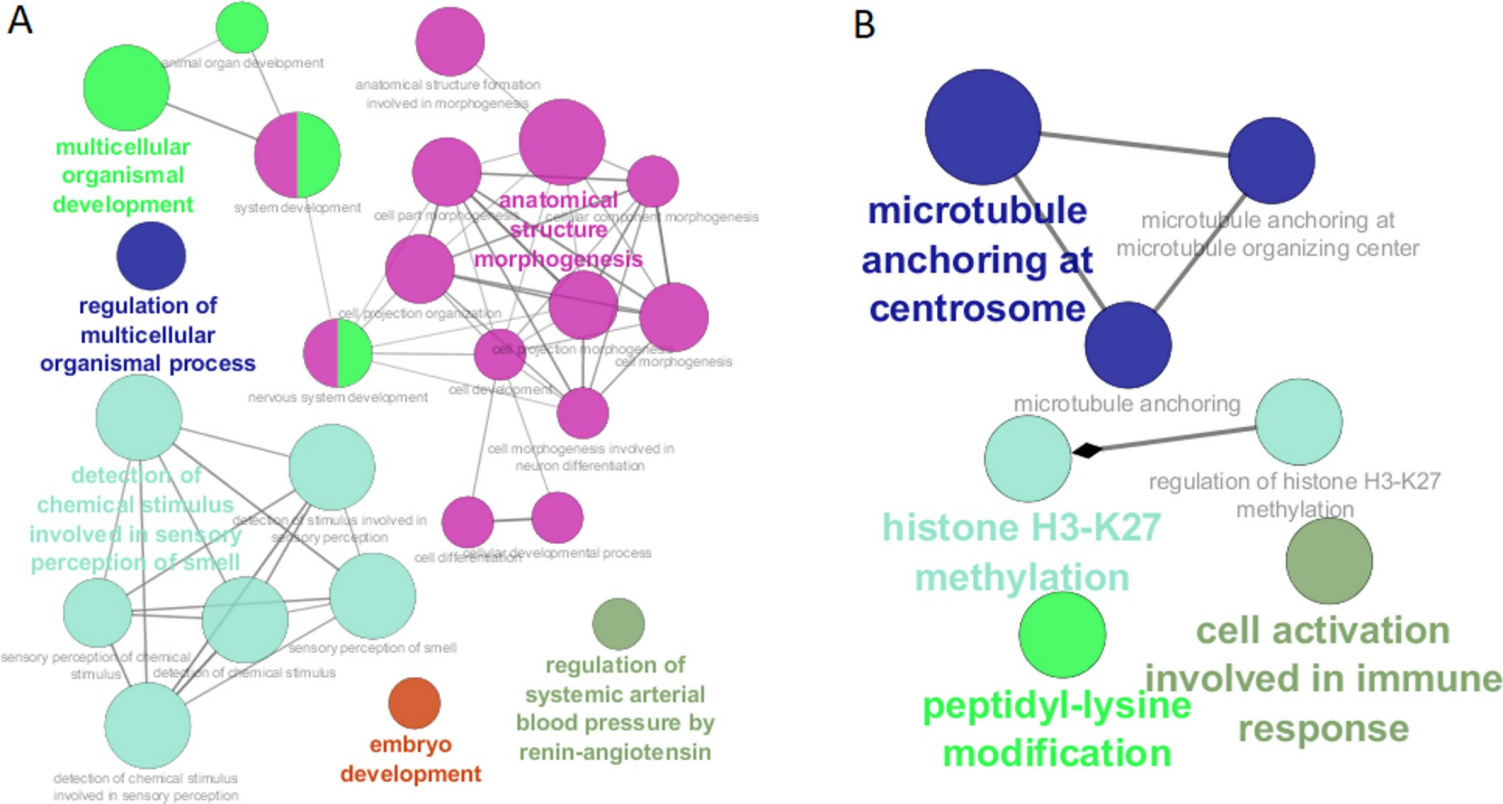
Biological processes affected by gene expression differences between both study groups of patients. A) underexpressed genes; B) overexpressed genes.

To present quantitative differences in the level of gene expression in the selected groups of patients, differential analysis of genes was performed. We calculated the relative difference in the expression of individual genes in clusters 1 and 2 compared to clusters 5 and 6. Genes for which the difference factor was lower than 1.3 were omitted in the further analysis under the assumption that they do not have a qualitative impact on the phenotype of the analysed patients. Consequently, we focused on those genes whose expression was the most diverse (differing by a factor of at least 1.3). In the following step, we analysed the processes in which those genes are involved. For the underexpressed genes, statistically significant involvement (p<0.05) was reported for the following processes (Fig 5A):

- regulation of systemic arterial blood pressure by renin-angiotensin
- embryo development
- morphogenesis of anatomical structures
- detection of chemical stimulus involved in sensory perception of smell
- multicellular organismal development
- regulation of the multicellular organismal process

In contrast, overexpressed genes were found to participate in the following processes (Fig 5B):

- microtubule anchoring at the centrosome
- histone H3-K27 methylation
- cell activation involved in immune response
- peptidyl-lysine modification

It is important to note that the processes in which differentiated genes are involved may be important for the progression of multiple myeloma. For example, genes whose protein products are associated with embryonic development undergo incorrect regulation during the development of myeloma, thus contributing to the progression of this cancer [20]. Particularly noteworthy is the renin-angiotensin system, which in classical terms is responsible for pressure regulation [21]. However, recent studies indicate that deregulation of the renin-angiotensin system is observed in some diseases, including cancer [22]. Changes in the expression of genes related to the renin-angiotensin system are observed in parental tumour cells, which indicate their importance in the process of carcinogenesis [23]. Among the overexpressed genes, a group associated with the centrosome was demonstrated. This may be important for the prognosis for multiple myeloma since previous studies have shown that the expression of genes associated with centrosomes is an independent predictor of myeloma patients [24]. The lysine 27 methylation process in histone 3 is a modification associated with gene repression and plays a key role in regulation that ensures a balance between differentiation and proliferation [25]. Accordingly, any aberrations associated with methylation of lysine 27 in histone 3 may translate into functional changes of myeloma cells, leading to the progression of this cancer. Based on these few examples, it can be seen that the processes which involve the differentiated genes are important in the development of myeloma, and that this knowledge may lead to therapies which target intensified or inhibited processes for specific groups of patients, as appropriate.

#### Comparison of GCA versus CUR matrix decompositions and random results

To evaluate the GCA method described in the manuscript, the results obtained by the GCA method were compared with the alternative method, i.e. CUR decomposition matrix and with a random data set. For this purpose, based on the CUR methodology, a subset of samples (genes) being representants of the original set was generated (k = 4). A random subset of samples (genes) was independently generated.

The subsets were grouped by the GCA method on six clusters (similarly to the original set) and then compared the overlapping samples (genes) in clusters.

**Fig 6:**
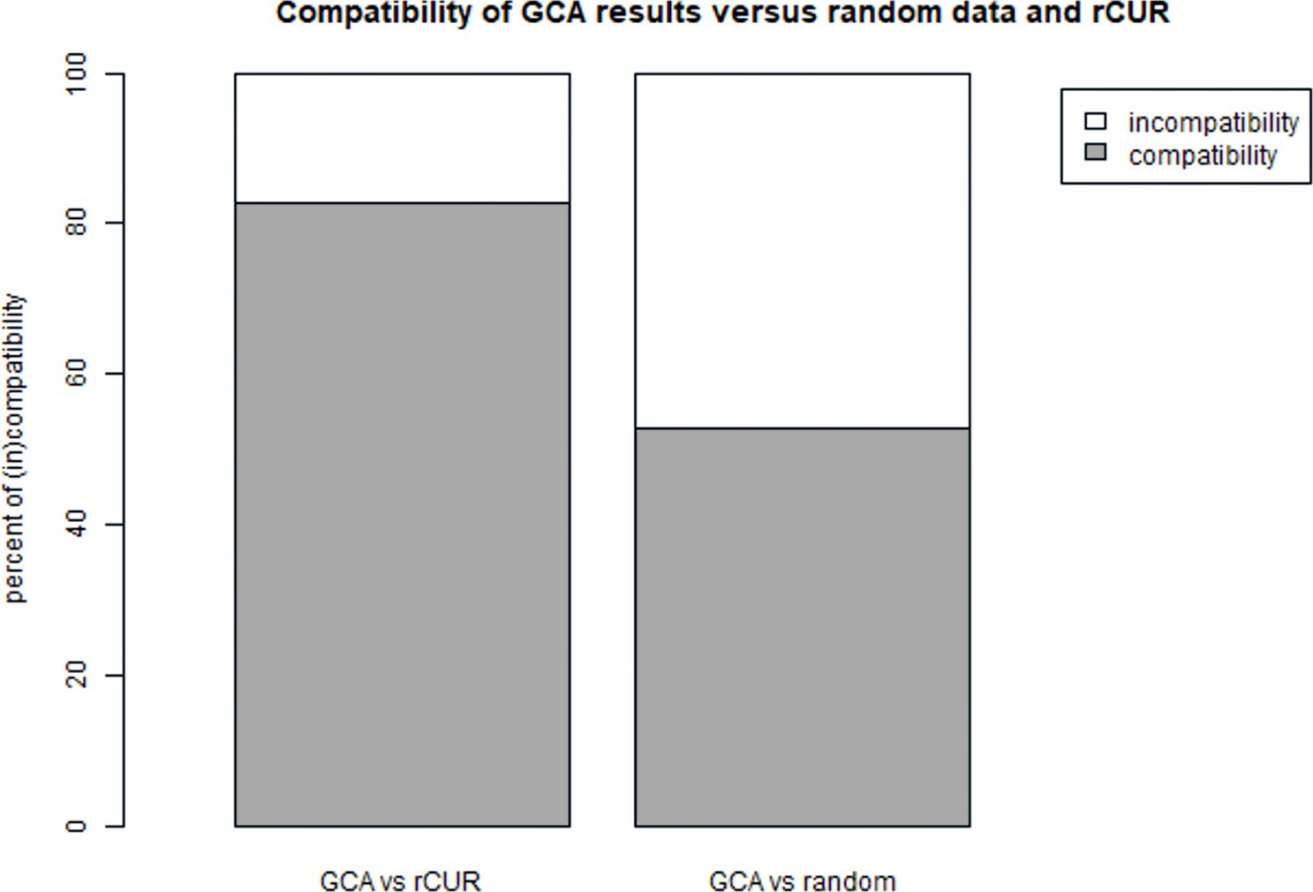
Comparison of GCA versus CUR matrix decompositions and random results.

The results were in the range of 72% -86% of compatibility of samples in clusters depending on the cluster for GCA and rCUR results. GCA results in comparison to CUR results for clusters 1 and 6 (from UP and DOWN regulated genes) showed compliance at the level of nearly 82% (Fig 6). In the case of the comparison of GCA results with the randomly selected samples (genes), compatibility was in the range of 43%-52%. For Cluster 1 and 6, the result was 52.9% compatibility and 47.1% non-compatibility (Fig 6).

Without penetrating the biological interpretation of the obtained results it was carried out analyzes on various data sets. Similar results of the method's effectiveness were obtained. In the case of data from the GDS2771 (Large airway epithelial cancer from suspect lung cancer) experiment, that the sorting of cancer data for GCA vs rCUR gives nearly 86% similar results while the case of GCA vs randomly "sorted" received compliance level: 46%. Based on a set derived from the GDS4337 (Type 2 diabetic and hyperglycemic pancreatic islets) experiment, narrowing the analysis to non-diabetic patients, the similarity of the obtained results: GCA vs rCUR -> 84%; GCA vs random -> 51%

## Conclusion

The Grade Correspondence Analysis method is an example of such an approach to an analysis of gene expression, which includes all probes and treats them independently. In gene expression microarray studies, hundreds of thousands of probe expressions are measured for a large number of samples. Not every probe for a particular gene gives a proportional result. Some probes show that a given gene has a higher expression, other lower. In a typical research methodology, the result for individual genes is the averaging of results obtained for all probes of a given gene. The publication of studies with dissimilar or contradictory results has raised concerns about the reliability of this way of analysis.

The Grade Correspondence Analysis is a method that can be overcome this problem. Itis an exploratory method which reveals hidden information by sorting all probes and all patients. By computing overrepresentation coefficients, the presented method can reveal the degree of discordance between the expected and observed values, assuming that the distribution remains perfectly proportional. The ordering of columns may be thought of as a representation of their relative “importance” for the structure of the data. Edge cases (located on either side of the sorted matrix) are more strongly indicative of the observed trends than items in the middle. Based on the Multiple Myeloma example it was shown how to distinguish groups of patients and sets of probes (being representatives of genes), to identify particularly interesting patients and genes that can be used as a starting point for further studies.

GCA and GCCA can be used as an alternative to popular data grouping methods [26] [27], e.g. gene expression profiles. Processing data with the GCA algorithm may provide an important step in the analysis of various biological processes. GCA results integrated with another biological data (e.g. biochemical pathways, protein interactions, gene signatures) [28] [29] [26] may constitute a valuable tool in the analysis of the interesting processes. The Grade Correspondence Analysis may be used to regularize and sort matrices including large *-omics* arrays (not only transcriptomics data).

In the case of large data resources (including large *-omics* arrays), to maintain a maximum amount of information, GCA method used to regularize and sort matrices seems to be a useful tool for analysis.
